# Application of mass spectrometry-based proteomics for biomarker discovery in neurological disorders

**DOI:** 10.4103/0972-2327.48845

**Published:** 2009

**Authors:** Abhilash Venugopal, Raghothama Chaerkady, Akhilesh Pandey

**Affiliations:** 1 Institute of Bioinformatics, Bangalore 560 066, India,; 2 McKusick-Nathans Institute of Genetic Medicine and Departments of Biological Chemistry, Oncology and Pathology, Johns Hopkins University, Baltimore, Maryland 21205, USA

**Keywords:** Biomarkers, diagnosis, isotope labeling, mass spectrometry

## Abstract

Mass spectrometry-based quantitative proteomics has emerged as a powerful approach that has the potential to accelerate biomarker discovery, both for diagnostic as well as therapeutic purposes. Proteomics has traditionally been synonymous with 2D gels but is increasingly shifting to the use of gel-free systems and liquid chromatography coupled to tandem mass spectrometry (LC-MS/MS). Quantitative proteomic approaches have already been applied to investigate various neurological disorders, especially in the context of identifying biomarkers from cerebrospinal fluid and serum. This review highlights the scope of different applications of quantitative proteomics in understanding neurological disorders with special emphasis on biomarker discovery.

## Introduction

Neurological disorders are a major cause of physical disability and mortality worldwide. However, there is still a paucity of studies investigating the molecular mechanisms of disease progression and biomarkers of diagnostic and prognostic value. Mass spectrometry-based proteomic profiling has been employed in investigating several neurological diseases[1–3] and to identify diagnostic biomarkers in psychosis, Guillain-Barré syndrome, multiple sclerosis and Alzheimer's disease.[4–9]

Proteome is the entire complement of proteins expressed by the genome at any given time in a cell, tissue or an organism, while proteomics deals with the characterizing features of gene products such as post-translational modifications, protein isoforms, subcellular localization, protein-protein interactions and tissue expression.[10,11] As proteins are the functional molecules in cells, measurement of the differential expression of proteins could indicate disease-specific changes in tissues or organs. *In vivo* labeling using the SILAC method or *in vitro* labeling approaches such as isotope-coded affinity tagging allows simultaneous identification of proteins and quantitation of their abundance levels.[12,14] A number of studies have shown that quantitative proteomics is a promising approach for discovery of potential biomarkers for early detection and to understand drug responses and molecular pathogenesis.[15] Candidate biomarkers identified using proteomic profiling of serum and cerebrospinal fluid (CSF) could be used for diagnosis, prognosis and determining therapeutic response to different treatment modalities.[1,4,16,17]

The majority of published studies by proteomic approaches to study neurological disorders have used two-dimensional gel electrophoresis (2-D PAGE), which has a number of limitations.[18–19] Quantitative mass spectrometry approach offers an attractive option to investigate disease-specific changes with high-degree of specificity and sensitivity. This brief review will present different types of quantitative proteomic approaches and their applications in neurological disorders.

## Trascriptomics versus proteomics

Genetic variability is plausible for different disease phenotypes; this could be at the level of alterations at transcription, translation and posttranslational modification of gene expression. DNA microarrays allow cataloging of gene expression under different conditions. Blalock *et al.* carried out a transcriptomic analysis of incipient Alzheimer's disease (AD) using DNA microarrays.[20] They studied gene expression profile in hippocampus of 9 control and 22 AD subjects of varying severity. The study revealed activation of growth and differentiation pathways, and downregulation of protein transport machinery. In the DNA microarray approach, the mRNAs are labeled with fluorescent dyes followed by hybridization with DNA probes immobilized in an array format at a very high density. Relative fluorescence between samples provides a measure of the relative abundance of mRNAs present in the samples. Table 1 outlines some of the considerations for sample collection and handling for mRNA and protein-based biomarker discovery. DNA microarrays provide readout of the transcriptional activity of genes but do not provide data on protein expression or post-translational modifications of proteins in the samples. Proteomic approaches, especially those involving mass spectrometry, provide data on protein expression as well as post-translational modifications in different disease conditions, which could lead to the discovery of biomarkers. The biomarker discovery pipeline using proteomics entails sample extraction, differential labeling of samples, fractionation, tandem mass spectrometry (MS/MS) and data analysis. To identify biomarkers in traumatic brain injury patients, Hergenroeder *et al.* used pooled sera from patients and labeled the samples using isobaric tags followed by liquid chromatography tandem mass spectrometry (LC-MS/MS) analysis.[21]

**Table 1:** A clinical guide for sample handling and storage for biomarker discovery

	RNA	Protein
**Samples**	Tissue and peripheral blood samples are widely used for RNA analysis (e.g. PCR, DNA microarrays)	Serum, plasma, tissue, CSF and other body fluids are normally used for proteomic analysis
**Sample handling**	Instruments and handling area should be sterile and free of RNase. Use solutions with RNase inhibitors (e.g. RNAzap).	Contamination with keratins from skin and hair should be avoided. Use gloves.
**Sample storage** and transport	Tissue should be either flash frozen or immersed in a tissue stabilization solution such as RNAlater.	**Tissues:** Tissues should be snap frozen in liquid nitrogen and stored at −80°C until used
	Archived frozen tissue should be quickly disrupted or solutions such as RNAlater-ICE should be used for thawing purposes. Multiple freeze/thaw cycles should be avoided. Immediate processing will minimize RNA degradation	**CSF:** Specimens should be centrifuged immediately at 2000g for 10 min at 4°C to remove cells and other debris. Supernatants should be snap frozen immediately and stored at −80°C in aliquots.
		**Serum/plasma:** The blood samples should be centrifuged at 1300 *g* at 4°C and stored at −80°C in aliquots.
Quality control	Quality of the samples should be judged by RNA purity (A_260_/A_280_) and integrity (18S:28S ratio).	The sample integrity can be assessed by SDS-PAGE.

## Mass spectrometry for proteomic analysis

Liquid chromatography coupled with tandem mass spectrometry (LC-MS/MS) is widely used today for characterization of biological samples with high level of sensitivity and specificity. Different mass spectrometric methods are available for proteomic profiling and identification of biomarkers. One of the platforms is surface-enhanced laser desorption-ionization (SELDI), which has been used to obtain disease specific proteomic patterns.[22] However, in this approach, only mass spectrometry peak patterns are obtained and the exact identity of the peaks are not determined (i.e. the proteins are not identified in this type of mass spectrometry).[23] Other platforms such as tandem mass spectrometry permit actual identification of amino acid sequences of peptides and are preferable to SELDI for detecting biomarkers.

There are several labeling approaches for performing mass spectrometry-based quantitative proteomics analysis. These include labeling methods such as stable isotope labeling with amino acids in cell culture (SILAC)[12] and isobaric tags for relative and absolute quantitation (iTRAQ),[24] cysteine labeling using isotope-coded affinity tags,[14] labeling with isotopically labeled acrylamide and C-terminal labeling using ^18^ O-labeled water.[25]

In a SILAC experiment, cells representing different biological conditions are grown in media supplemented with either “light” or “heavy” isotope form of amino acids. In this method, labeled amino acids are metabolically incorporated into all peptides and subsequent pooling of differentially labeled samples in equal ratios provides quantification of peptides from each sample. This quantification of proteins is based on the relative intensities of corresponding differentially labeled peptides. SILAC has been used to study signaling in several systems including the phosphorylation dynamics of ion channels and to evaluate the brain derived neurotrophic factor (BDNF) induced change in neuronal phosphotyrosine proteome.[26,27] However, the disadvantages of this method is that, it cannot be used for tissues or body fluids and might require further validation as the experiments are carried out in cell lines. Park *et al.* used this method to quantitate the phosphorylation of Kv2.1 protein, which was transfected to HEK293 cell lines and activated by ionomycin to study calcineurin dependent dephosphorylation.[26] It has also been used to identify BDNF-induced proteins, which includes *tyrosine kinase receptor B, hepatocyte growth factor-regulated tyrosine kinase substrate* and signal transducing adaptor molecule. All these proteins are known to control the molecular trafficking of receptor tyrosine kinases.[27]

**Figure 1:**
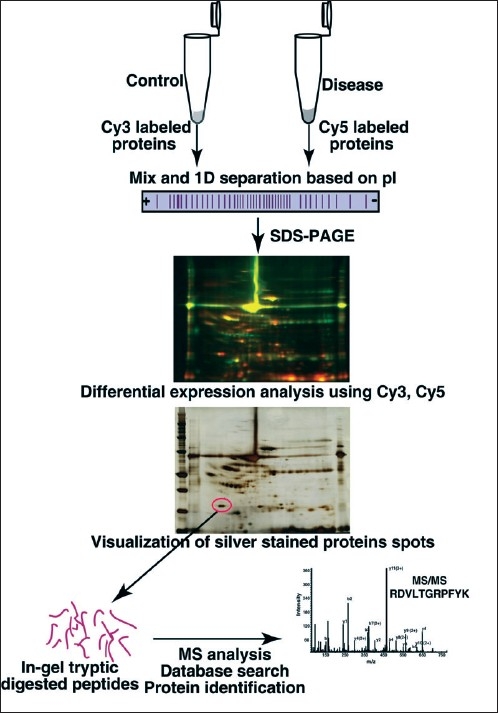
2D-DIGE based separation of proteins and identification by mass spectrometry. Proteins from two samples are labeled using Cy3 or Cy5 prior to pooling. First dimension separation of proteins is carried out using immobilized pI gradient strip and second dimension separation is carried out on SDS-PAGE gel. Differentially expressed proteins are identified using a fluorescence scanner and unique spots are subjected to in-gel trypsin digestion, released peptides could be analyzed on LC-MS/MS for further identification

2D-PAGE is widely used for protein separation for comparative proteomic profiling [Figure 1]. DIGE is a vital part of the 2D-PAGE for protein quantitation,[28] in spite of its limitations (the biggest limitation is that only the most abundant proteins can be analyzed).[29] In DIGE, different samples are labeled with different fluorescent dyes prior to separation, facilitating analysis of diversity in protein abundance between samples. DIGE separates protein samples into spots, which could be subsequently identified by tandem mass spectrometry. Software is required to extract quantitative information; additional normalization[30] and stringent statistical tests are often required for analyzing the data.[31] The data obtained from the 2D gel analysis should be validated using immunohistochemical labeling or enzyme assays, as the protein spots in 2-D gel can co-migrate leading to confusion in the identity of proteins. Some of the neurological disease biomarkers revealed by DIGE approach are listed in Table 2. Gao *et al.* performed DIGE to analyze the proteomic profile of cerebrospinal fluid in traumatic brain injury in infants, which is one of the common causes of mortality in infancy. The study identified candidate biomarkers such as *prostaglandin D2 synthase and cystatin C,* further confirmed by Western blot.[19] Maternal serum profiling was performed to identify candidate biomarkers for Down syndrome using 2D-DIGE coupled to MALDI-TOF MS analysis.[18] This study proposed differential expression of molecules such as *afamin, alpha-1-microglobulin, apolipoprotein E* and *ceruloplasmin* in the maternal serum.

**Table 2:** Published studies using quantitative proteomic methods for identification of biomarkers in neurological disorders

Quantitative proteomics method	Neurological diseases	Features	Tissues used	Potential biomarkers	Reference
2DE	Huntington's disease.	Combination of MALDI-TOF and LC-MS/MS platforms for biomarker discovery	Plasma	Clusterin, alpha 2 macroglobulin.	Dalrymple *et al*.[45]
2D-DIGE	Down syndrome	Proteomic analysis of maternal serum	Serum	Alpha-1-acid, glycoprotein 1.	Nagalla *et al*.[18]
2D- DIGE	Guillain-Barré syndrome	Differential proteomic profiling MALDI-TOF MS	CSF	Haptoglobin, Apolipoprotein A-IV.	Yang *et al.*[55]
SDS-PAGE	Multiple sclerosis	Capillary liquid chromatography-electro spray-ion trap mass spectrometry.	CSF	CRTAC-IB, tetranectin.	Hammack *et al*.[56]
2D-DIGE	Traumatic brain injury of infancy	Western blot confirmation of the identified biomarkers	CSF	Cystatin C, PGDS.	Gao *et al*.[19]
2DE	Alzheimer's Disease	Differential proteomic profiling	CSF	Cathepsin-D, Apolipoprotein A-I.	Castano *et al*.[57]
6-plex isobaric labeling	Brain injury	MALDI TOF/TOF and ESI-Q-TOF	CSF	GFAP, protein S100B, PARK7.	Dayon *et al.*[58]
SDS-PAGE	Multiple sclerosis	Laser-capture micro dissection	Brain	Tissue factor, protein C inhibitor.	Han *et al.*[59]
ICAT	Alzheimer's disease	LC-MS/MS	CSF	Amyloid precursor protein, Cathepsin B.	Zang *et al.*[60]
Capillary isoelectric focusing	Glioblastoma multiformae	LC-MS/MS	Brain cortex	Wolf-Hirschhorn syndrome candidate 1.	Li *et al.*[61]
2DE	Systemic lupus erythematosus	LC-MS/MS	Serum	Autoantigens for HSP90	Kimura *et al*.[62]
8-plex iTRAQ	Alzheimer's disease	SCX and LC-MS/MS	CSF	Clusterin, hemopoxin	Choe *et al.*[32]
iTRAQ	Traumatic brain injury	LC-MS/MS	Serum	Serum amyloid A, C-reactive protein	Hergenroeder *et al.*[21]
2DE	Traumatic brain injury	MALDI-TOF	CSF	Alpha 1 antitrypsin, haptoglobin 1 alpha 1.	Conti *et al.*[47]

**Table 3:** Application of mass spectrometry-based proteomics to study neurological diseases

Neurological diseases	Tissues used for proteomic analysis	Clinical use
Neoplasms of the central nervous system	CSF/Serum	Early, relatively noninvasive diagnosis and monitoring of disease progression
Multiple sclerosis	CSF/Serum	Identify disease progression and response to therapy
Neuro-degenerative diseases	CSF/Serum	Differentiate between diseases with similar initial clinical presentation
Motor neuron disease	CSF/Serum	Distinguish motor neuron diseases from multifocal motor neuropathy
Meningitis	Serum/CSF	Early diagnosis and monitoring of therapy
Encephalitis	Serum/CSF	To distinguish different etiology of encephalitis
Psychosis	Serum/CSF	To distinguish different etiologic forms of psychosis and to monitor the therapy

iTRAQ reagents are a set of isobaric tags that bind to primary amines by covalent bond leading to the labeling of peptides. Measuring the relative intensity of these reporter ions in MS/MS spectra allows the relative quantitation of the proteins in samples. Choe *et al.* used 8-plex iTRAQ quantitation of proteins in cerebrospinal fluid of the patients with Alzheimer's disease. The study observed a decrease in some proteins, which include albumin, *clusterin* and *hemopoxin.*[32] The proteins with the increased expression include *apolipoprotein E* and *cystatin C*. Since eight types of the reporter molecules are now available, this method allows multiplexing of up to eight sets of samples in a single mass spectrometry experiment. Table 2 provides the list of some of proteomic studies in neurological diseases. Hergenroeder *et al.* investigated for candidate serum biomarkers for brain injury using iTRAQ method coupled with LC-MS/MS. The study identified *serum amyloid A, C-reactive protein* and *retinol binding protein 4* and these candidate biomarkers were verified by enzyme-linked immunosorbent assay (ELISA) in an independent set of serum samples from cases of traumatic brain injury and healthy volunteers.[21]

## Biomarker discovery in CSF and serum – promises and challenges

One of the major goals of diagnostic biomarker discovery is development of a simple and accurate blood test for early diagnosis of disease. Human serum/plasma remains the easily accessible and commonly used clinical sample for proteomics applications. Human plasma has been described as a circulating representation of all body tissues in both physiological and pathological processes.[33] This is because serum/plasma is in direct or indirect contact with the cells or tissues in the body hence may contain specific biomarkers in soluble phase for easy identification. However, the advantages of plasma as one of the easy to obtain sample is counter balanced by analytical challenges posed by the complexity of the plasma proteome and also the genetic diversity of human population. Sample preservation for proteomics analysis also plays a crucial role in outcome of quantitation.

Pre-analytical variables have a potential influence on the accuracy and reproducibility of quantitative proteomic analysis. These variables include the type of collection tube used, the clotting time, sample transit time, storage conditions and use of protein inhibitory cocktails. These differences are driven by proteolysis with other factors, such as agglutination or differential adhesion of serum polypeptides to the walls of the tube. For serum analysis, the sample should be allowed to clot for 30–60 min before processing to minimize the differences arising from the effects of coagulation.[34] Plasma preparation for proteomic analysis is carried out by treating the samples with anticoagulants, which also have inherent advantages and disadvantages.[35] The choice of the anticoagulants, such as EDTA, citrate and heparin, is based on the downstream applications of the sample. Other pre-analytical variables such as centrifugation (speed, time, and temperature) are likely to have significant effect on sample stability. The use of protein inhibitor cocktails for storage of body fluids has detectable benefits in stabilizing the proteins. Another factor that affects the proteomic analysis is storage temperature.[35] The samples should be stored frozen and repeated freeze/thaw cycles should be avoided and samples should be subjected to uniform protocol of handling for reproducible quantitative analysis.

**Figure 2:**
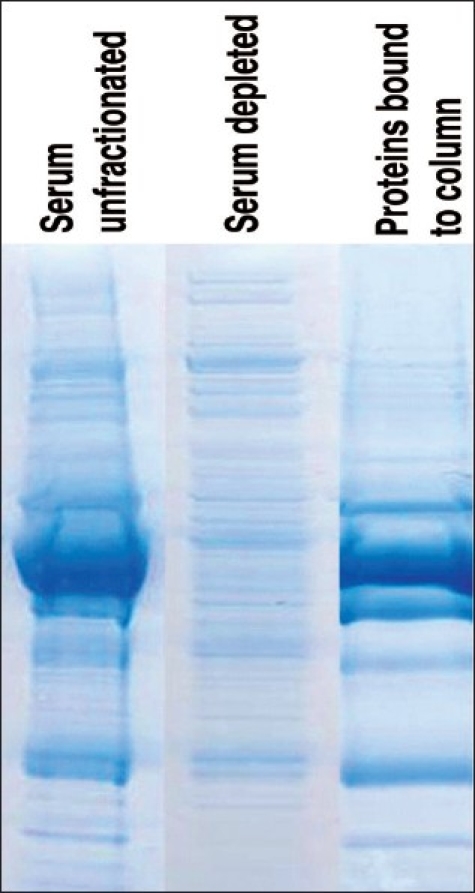
Multiple affinity depletion of serum proteins. Serum from a normal individual was separated using multiple antigen affinity removal column (from Agilent Technologies). Lane 1, Whole serum. Lane 2, Unbound fraction representing depleted serum. Lane 3, Proteins depleted by affinity column. Note the change in number of bands and their density

To optimize the sensitivity limits of biomarker discovery using mass spectrometry, enrichment of less abundant proteins in serum (proteins present in subtle quantities and yet important in physiology) is critical – this might require the depletion of the abundant proteins such as albumin, immunoglobulins, transferrin and macroglobulins. Depletion of these abundant proteins can be carried out by affinity chromatography methods involving multiple protein depletion in a single separation step. This increases the chances of detecting candidate biomarkers, which are likely to be present in low abundance. Figure 2 demonstrates the effect of depletion of major six proteins from serum using a multiple affinity column (Agilent, Multiple Affinity Removal LC Column). Ramstrom *et al.* reported a method for removal of “brain-specific abundant protein” in addition to common serum proteins.[36] The damage to the blood-brain barrier (BBB), a known event in many of the neurological diseases, may enhance movement and mixing of proteins between the brain interstitial space and blood; thus serum can be considered as an appropriate sample to study.[37] However, since the CSF provides a direct insight into the neurological microenvironment, it should be considered a preferred body fluid for proteomic analysis for biomarker discovery and diagnosis of neurological diseases.[9] In pathological states, the cells in the brain are likely to shed the altered proteins into CSF and thus be available for analysis.[38] Apart from this, the high amount of immunoglobulins in many neurological diseases also indicates the need for specific isolation and fractionation methods.[10,39] Using multiple affinity purification columns, majority of the abundant proteins from serum and CSF can be depleted before the proteomics analysis.[40]

## Mass spectrometry to identify biomarkers for early diagnosis of neurological diseases

Many neurological diseases are analyzed for identification of candidate biomarkers by different proteomic approaches. Here, we provide an overview of published studies in which proteomic profiling was used to understand various neurological disorders. Table 3 lists some of the applications of mass spectrometry-based proteomics in different neurological diseases.

### Psychosis:

Proteomic profiling of cerebrospinal fluid from patients with first onset of psychosis, using SELDI mass spectrometry, identified increased *VGF-derived peptide* in cases of schizophrenia compared to healthy volunteers.[5] Further validation of *VGF-derived peptide* in an independent set of psychosis patients with schizophrenia and without schizophrenia could be useful to prove that the proteins identified are really schizophrenia specific. Craddock *et al.* profiled the proteome of T cell lysates from schizophrenia patients, and the study identified *alpha defensin* as a candidate biomarker, which was also verified in an independent set of patients of schizophrenia, their family members and healthy volunteers using ELISA.[1]

### Neurodegenerative diseases:

Sultana *et al.* analyzed hippocampus proteome of Alzheimer's disease patients.[41] The differentially upregulated proteins include *enolase, ubiquitin carboxyl terminal hydrolase L-1 and triosephosphate isomerase*. Simonsen *et al.* used cerebrospinal fluid to detect candidate biomarkers, which are capable to differentiate between patients with stable mild cognitive impairment (MCI) and those who will progress to Alzheimer's disease (AD).[42] The study identified proteins such as *ubiquitin and phosphorylated C-terminal fragment of osteopontin* as potential biomarkers. By quantitative proteomic analysis of frontal cortex of parkinsonism-dementia complex cases using iTRAQ labeling, *alpha synuclein* is identified as a candidate biomarker.[43] Lee *et al.* performed peptide mass finger printing to identify candidates for Alzheimer's disease associated proteins[44] and found *fibrinogen gamma-A chain precursor protein* to be upregulated with the progression of the disease. Profiling of plasma proteome in Huntington's disease revealed over expression of identified *clusterin* and *IL-6* with respect to controls.[45] Likewise, validation by ELISA experiments on an independent set of patient samples confirmed that *clusterin* and *IL-6* are increased in plasma. Stoop *et al.* used quantitative mass spectrometry to profile the cerebrospinal fluid proteome of multiple sclerosis patients where a panel of candidate biomarkers were identified such as *chromogranin A, clusterin, complement C3* and *complement C4B.*[8]

### Neurotrauma:

Haqqani *et al.* used isotope-coded affinity tags (ICAT) followed by tandem mass spectrometry to perform serum proteomic profiling of pediatric patients of traumatic brain injury, where serum samples were depleted of high abundant molecules such as albumin and immunoglobulin.[46] The proposed candidate biomarkers included *ornithine carbamoyl transferase, IL-1R like precursor* and *Toll like receptor 9 like precursor*. Conti *et al.* analyzed CSF from cases of severe traumatic brain injury using 2D-PAGE combined with mass spectrometry.[47] In this study, proteins such as *alpha 1 antitrypsin, haptoglobin 1 alpha1, alpha2*, and *beta* belonging to the acute phase response were found over expressed. Interestingly, two other proteins, identified as proteolytic degradation products of the *carboxyl-terminal portion of the fibrinogen beta*, were present in the CSF of individuals with traumatic brain injury. These candidate proteins could be indicators of post traumatic inflammatory process as well as the fibrinolysis in the microenvironment of the tissue injury.

### Neoplasms of the central nervous system:

Roy *et al.* used differential quantitation without isotopic labeling coupled to LC-MS/MS to profile the proteome of cerebrospinal fluid from CNS-lymphoma patients.[7] Here, the study elucidated candidate biomarkers such as *antithrombin III* and *alpha-1-acid glycoprotein.protein*. Additionally, the expression of *antithrombin III* was immunohistochemically validated in CNS lymphoma tissues and also found in cerebrospinal fluid by ELISA. For elucidating therapeutic biomarkers of angiogenesis in glioma, Mustafa *et al.* explored the proteome of glioma tumor vessels, which was microdissected by Laser Capture Microscopy.[2] The study employed nano-LC fractionation and MALDI-FTMS to identify the potential biomarkers including *fibronectin* and *colligin 2*. Further, *fibronectin* and *colligin 2* were validated on glioma tissue sections using specific antibodies. Park *et al.* used 2D-PAGE and MALDI-TOF mass spectrometry approach to analyze the proteome of anaplastic oligodendroglioma tissues, where *peroxiredoxin 6* was identified to be overexpressed and later validated by Western blot and immunohistochemistry.[48] Khwaja *et al.* identified potential biomarkers in cerebrospinal fluid for central nervous system malignancies using MALDI analysis.[49] The study identified *cystatin* and *carbonic anhydrase*, which needs be further validated for its sensitivity and specificity.

### HIV-associated cognitive impairment:

Cerebrospinal fluids from HIV-1 seropositive patients were studied using proteomics platform consisting of SELDI-TOF, reverse phase high performance liquid chromatography (RP-HPLC) sample fractionation, SDS-PAGE, and LC-MS/MS.[50] The study identified proteins such as soluble *superoxide dismutase (SOD1), migration inhibitory factor (MIF) –related protein 14, macrophage capping protein, neurosecretory protein VGF, galectin-7, L-plastin, acylphosphatase 1* and a *tyrosine 3/tryptophan 5-monooxygenase activation protein* as differentially expressed. Further, SOD1 was validated by Western blot analysis. The clinical relevance and application needs further study.

## Mass spectrometry for investigating mechanisms of neurological diseases

Proteomic analysis could help to understand complex protein-protein interactions and molecular pathways in physiological functions of the nervous system. Husi *et al.* characterized *N-methyl-D-aspartate receptors (NMDAR) multiprotein complex* by immunoaffinity chromatography followed by LC-MS.[51] *NMDAR multiprotein complexes (NRC)* comprise 77 different proteins, including receptors, adaptors, signaling molecules, cytoskeletal and novel proteins. NRC mediates long-lasting changes in synapse strength via downstream signaling pathways. Genetic or pharmacological interference with 15 NMDAR multiprotein complex proteins impairs learning, while with 22 proteins, it alters synaptic plasticity in rodents. Here, mutations in three human genes (NF1, Rsk-2, L1) are associated with learning impairments, indicating that the NRC also participates in human cognition. A study of synaptic multiprotein complexes associated with the *5-HT (2C) receptor* has also been carried out by affinity chromatography and 2D-PAGE followed my MALDI-TOF mass spectrometry.[3] This approach identified 15 proteins that interact with the C- terminal tail of the *5-hydroxytryptamine 2C (5-HT_2C_) receptor*, a *G-protein-coupled receptor*. These proteins include several synaptic multidomain proteins containing one or several PDZ domains (PSD95 and the proteins of the tripartite complex Veli3-CASK-Mint1), proteins of the actin/spectrin cytoskeleton and signaling proteins. Identification of these proteins could help in understanding the signaling pathways associated with *5-HT_2C_ receptors* in neurons. Activation of *5-HT_2C_ receptors* exerts a phasic and tonic inhibition of the mesocorticolimbic dopamine function. This suggests that *5-HT_2C_ receptor* antagonists may be useful for the treatment of negative schizophrenia symptoms.

A SELDI-based approach has been used for the development of potential markers for Stiff person syndrome (SPS).[52] This approach identified downregulation of *GABA-A-receptor-associated* protein *(GABARAP)*, which enables assembly of *GABAA-receptor* into the plasma membrane. The level of this protein was found to be inversely correlated with the autoantibodies raised against GABA-A-receptor-associated protein in the sera of patients, leading to the conclusion that *GABARAP* could be a new potential auto antigen in SPS, which could be damaging GABAergic pathway, pathogenetically involved in disease evolution. Analysis of phosphorylation in Kv1.2-containing Kv channels by SILAC labeling identified *in vivo* phosphoserine (pS) sites at pS434, pS440 and pS441.[53] The study suggests that stimuli that alter activity levels of Ser-directed protein kinases and phosphatases may affect expression levels of Kv1.2-containing Kv channels through an effects on C-terminal of Kv1.2 phosphorylation sites and biosynthetic intracellular trafficking. Changes in the Kv channel phosphorylation state mediate changes in electrical excitability in response to altered synaptic activity and thus neuromodulation leading to the susceptibility to seizure due to the reduced trafficking. Mass spectrometric analysis of isolated di-heteromeric receptors identified a novel *NMDAR interactor* and *collapsin response mediator protein 2 (CRMP2)* that preferentially associates with NR2B-containing di-heteromeric NMDARs.[54]

## Future perspectives

Biomarkers are essential for understanding the pathogenesis and development of diagnostic and therapeutic tools in complex neurological disorders. With the recent advances in mass spectrometry and labeling methods, quantitative proteomics holds immense potential for biomarker discovery of neurological disorders. Subproteome analysis by means of depletion of abundant proteins, lectin affinity fractionation, and subcellular fractionation may facilitate the identification of vital molecules by reducing the complexity. With a panel of candidate diagnostic biomarkers, validation can be performed on larger patient population using proteomic techniques such as bead-based assays. High throughput proteomic studies involved in global analysis of neurological disorders form a valuable repository of data and can be useful in translating effectively the discoveries into clinical practice. Neuroproteomics has an added advantage of correlating phenotypic changes such as altered cognition and learning with proteomic changes. In conclusion, mass spectrometry-based proteomics offers a promising platform to understand pathogenesis of disease and for discovery of biomarkers for early detection. A close interaction between the clinician and the specialist basic scientist will unravel new avenues of early diagnosis and evolving therapeutic strategies.[62]
